# Role of the C-Type Lectin Receptors MCL and DCIR in Experimental Colitis

**DOI:** 10.1371/journal.pone.0103281

**Published:** 2014-07-28

**Authors:** Julia Hütter, Magdalena Eriksson, Timo Johannssen, Robert Klopfleisch, Dorthe von Smolinski, Achim D. Gruber, Peter H. Seeberger, Bernd Lepenies

**Affiliations:** 1 Max Planck Institute of Colloids and Interfaces, Department of Biomolecular Systems, Potsdam, Germany; 2 Freie Universität Berlin, Institute of Chemistry and Biochemistry, Department of Biology, Chemistry and Pharmacy, Berlin, Germany; 3 Freie Universität Berlin, Department of Veterinary Pathology, Berlin, Germany; Duke University Medical Center, United States of America

## Abstract

Inflammatory bowel disease (IBD) is a chronic inflammatory disorder of the gastrointestinal tract. Though its exact etiology is still unclear, it is proposed that an imbalance in the intestinal homeostasis leads to a disturbed interaction between commensal microbiota and the mucosal immune system. Previous studies have shown that both innate and adaptive immunity are involved in an overwhelming colon inflammation, and thus contribute to the pathogenesis of IBD. In innate immunity, several pattern recognition receptors such as Toll-like receptors, NOD-like receptors or C-type lectin receptors (CLRs) are involved in IBD pathogenesis. Myeloid CLRs are mainly expressed by antigen-presenting cells and bind to glycan structures present on self or foreign antigens. The Macrophage-restricted C-type lectin (MCL) and the Dendritic cell immunoreceptor (DCIR) are two poorly characterized members of the CLR family. In this study, we investigated the role of MCL and DCIR in the pathogenesis of murine colitis. Both CLRs bound to intestinal microbiota to a different extent. They modulated the production of pro-inflammatory cytokines by antigen-presenting cells upon stimulation with heat-killed microbiota and impacted subsequent T cell responses. To analyze whether MCL and DCIR contribute to the pathogenesis of IBD, the dextran sulfate sodium (DSS) murine colitis model was employed. MCL^−/−^ as well as DCIR^−/−^ mice exhibited only a slightly increased severity of disease compared to wild-type mice indicating a limited role for MCL and DCIR in the regulation of intestinal immunity.

## Introduction

Inflammatory bowel disease (IBD) is a chronic inflammatory disorder of the gastrointestinal tract. In humans, there are two major clinical forms: ulcerative colitis (UC) and Crohn’s disease (CD) [1]. UC mainly affects the mucosa of the colon and rectum, whereas inflammation in CD may occur in any part of the gastrointestinal tract [2]. The incidence of IBD, particularly CD, has dramatically increased in industrialized nations over the last decades, however, the exact etiology of IBD still remains unclear [3]. Several factors were reported to play a role in IBD pathogenesis such as environmental factors, diet, genetic factors and the immune system [1]. These factors can influence the composition of commensal microorganisms in the intestine followed by an imbalanced intestinal homeostasis which is a major characteristic feature of IBD [4].

Pattern recognition receptors (PRRs) expressed by antigen presenting cells (APCs), especially dendritic cells (DCs), function as receptors for endogenous as well as pathogenic antigens [5]. In a healthy gut, DCs sample intestinal antigens with the help of PRRs, internalize them, migrate to local lymphoid tissue and subsequently present the processed antigens to T cells [6]. Thus, PRRs are essential for DCs to maintain intestinal immune homeostasis [6]. Based on their physiological function, several PRRs such as NOD2, NLRP3 and several Toll-like receptors (TLRs) were demonstrated to influence the pathogenesis of IBD [7]–[9]. Several TLR-deficient mice or mice lacking the adaptor protein MyD88 exhibited an increased susceptibility to experimental colitis induced by oral administration of dextran sodium sulfate (DSS) [8], [10]. The DSS model is commonly used to investigate the contribution of innate immunity to the pathogenesis of IBD [11], [12]. An increased susceptibility to DSS-induced colitis was also reported for mice deficient for the cytosolic NOD-like receptors (NLRs) NOD1 and NOD2 [9].

C-type lectin receptors (CLRs) recognize various classes of pathogens [13]–[15]. They are mainly expressed by antigen-presenting cells such as monocytes, macrophages, and DCs and recognize carbohydrate moieties on pathogens as well as on self-antigens [16], [17]. Many CLRs such as DC-SIGN, SIGNR3, Dectin-2, MGL or Langerin are internalized after ligand binding followed by processing of the antigen and subsequent MHC class II presentation or cross-presentation to T cells [16]. Previous studies indicate that CLRs play a crucial role in the regulation of intestinal immune homeostasis and colon inflammation [18]. A polymorphism in the human Dectin-1 gene (CLEC7A) is associated with a severe form of UC [19]. Consistently, mice deficient in Dectin-1 exhibited increased susceptibility to DSS-induced colitis due to an altered response to commensal intestinal fungi [19]. The Macrophage galactose-type lectin-1 (MGL1) plays a regulatory role in murine colitis as it contributes to IL-10 secretion by lamina propria macrophages upon interaction with invading commensal bacteria [20]. Mice deficient for the murine DC-SIGN homologue SIGNR1 exhibited a reduced susceptibility to experimental colitis since SIGNR1 acts synergistically with TLR4 in the initiation of an inflammatory response upon LPS binding by TLR4 [21]. Recently, it was demonstrated that the murine CLR SIGNR3 plays a protective role in chemically induced colitis in mice [22]. A number of studies focused on the role of the Mannose-binding lectin (MBL) in intestinal inflammation. Mice deficient for MBL exhibited an increased susceptibility to DSS-induced colitis and MBL in humans was reported to ameliorate the excessive inflammation during IBD [23], [24].

Two poorly characterized CLRs are the Macrophage-restricted C-type lectin (MCL) and the Dendritic cell immunoreceptor (DCIR). MCL is a type II transmembrane glycoprotein with a single extracellular C-type lectin domain and is expressed by myeloid cells, mainly resting macrophages [25], [26]. The human MCL was found to be rapidly endocytosed upon receptor cross-linking [26]. Recently, it has been reported that (rat) MCL forms a heterodimer with the macrophage inducible C-type lectin (Mincle) [27]. MCL recognizes the mycobacterial glycolipid trehalose-6,6-dimycolate (TDM) which is a natural ligand of Mincle [28], [29] but no further ligands of MCL were identified up to now. A recent study indicated that MCL acts as an activating receptor that mediates phagocytosis, respiratory burst, and inflammatory cytokine production [30]. In addition to its role in mycobacterial infection, MCL is also involved in the immune response against *Klebsiella pneumoniae* infection [28], [31].

DCIR is expressed by DCs, B cells, monocytes, macrophages, and neutrophils [32] and was found to be internalized upon binding of a DCIR-specific antibody [33]. Furthermore, DCIR targeting allowed for antigen processing and presentation for efficient CD4^+^ as well as CD8^+^ T cell priming [34], [35]. DCIR is the only classical CLR with an intracellular immunoreceptor tyrosine-based inhibitory motif (ITIM) [36]. Upon activation, it recruits SHP-1 and SHP-2 phosphatases which subsequently promote the inhibition of ITAM signaling, particularly of Syk-coupled CLRs [16]. Based on its function, DCIR is down-regulated upon activation of DCs and plays an inhibitory role in the response to pathogens [32], [36].

In the present study, we investigated the role of MCL and DCIR in murine experimental colitis. Binding studies using CLR-hFc fusion proteins showed that MCL and to a low extent DCIR bound to commensal intestinal microbes. Furthermore, stimulation of MCL- and DCIR-deficient APCs with heat-killed commensal microbiota revealed a modulatory role of both CLRs in the production of pro-inflammatory cytokines. To analyze the role of MCL and DCIR in intestinal immunity *in vivo*, the murine DSS colitis model was employed. Local MCL mRNA levels in the colon were increased during experimental colitis. However, MCL^−/−^ as well as DCIR^−/−^ mice exhibited only a slightly increased disease severity based on either clinical symptoms or histopathology compared to wild-type mice indicating a limited role for MCL and DCIR in the regulation of intestinal immunity.

## Materials and Methods

### Generation of bone-marrow derived macrophages and dendritic cells

For cell stimulation studies, bone marrow-derived macrophages (BMMs) or bone marrow-derived dendritic cells (BMDCs) were generated from C57BL/6 J wild-type, DCIR^−/−^ (031932-UCD, DCIR KO/Mmcd), and MCL^−/−^ (031935-UCD, MCL KO/Mmcd) mice. Bone marrow cells were prepared from murine hind legs by flushing the uncovered and opened femurs and tibiae with medium. Red blood cells were lysed by addition of a buffer containing 144 mM ammonium chloride and 10 mM Tris-HCl pH 7.2 for 5 min. Subsequently, cells were separated with a cell strainer and cultivated in full IMDM medium (10% FCS, 200 mM L-glutamine, 10.000 U/mL penicillin, 10 mg/mL streptomycin) supplemented with 5% horse serum and 30% L929 cell supernatant for generation of BMMs or with 10% culture supernatant of GM-CSF transfected X-63 cells for generation of BMDCs [37]. The culture medium was replaced every two or three days and the cells were used for stimulation studies between day 7 and day 9.

### Binding studies with CLR-hFc fusion proteins

Analysis of commensal microbiota recognition by distinct CLRs was performed by incubating respective hFc fusion proteins with murine faecal samples. The extracellular parts of MCL, DCIR, and MGL1 were expressed as hFc fusion proteins as described previously [38]–[40]. Faecal samples were isolated from colon of female C57BL/6 J mice, filtered through a 40 µm cell strainer (BD Biosciences, Heidelberg, Germany), adjusted to an OD_600_ of 0.6 with sterile PBS and inactivated at 65°C for 2 h. Commensal microbes present in the samples were labeled with SYTO 61 fluorescent dye (Life Technologies, Darmstadt, Germany) at 2.5 µM in PBS for 30 min. CLR-hFc fusion proteins were added to the microbiota samples at 1 µg/mL in lectin buffer (50 mM HEPES, 5 mM CaCl_2_, 5 mM MgCl_2_, pH 7.4) supplemented with 0.5% (w/v) BSA at 4°C for 1 h. As a negative control, the hFc fragment alone was used. Bound fusion proteins were detected using PE-conjugated anti-hFc antibody (Dianova, Hamburg, Germany), diluted 1∶100 (v/v), at 4°C for 30 min. Gram-positive intestinal bacteria were detected by co-staining with the lectin wheat germ agglutinin (WGA)-AF 647 (Life Technologies) at 1 µg/mL that recognizes *N*-acetylglucosamine (GlcNAc) moieties [41]. Samples were washed three times after each staining step and subsequently analyzed by flow cytometry using a FACS Canto II flow cytometer (BD Pharmingen, Heidelberg, Germany). The FlowJo (Tree Star Inc., Ashland, OR) software was used for data analysis.

### Stimulation studies with commensal intestinal microbes

For cell stimulation with intestinal microbiota, murine faeces were prepared as described above. For direct stimulation of APCs, 1×10^5^ BMMs or BMDCs per well were seeded in a flat-bottom plate in full IMDM medium and were stimulated with various dilutions of faecal samples for 18 h. As positive control, cells were incubated with 1000 ng/mL LPS (Sigma Aldrich, St. Louis, MO) or plate-coated zymosan (Imgenex, San Diego, CA). The next day, cytokine concentrations in the culture supernatants were measured by ELISA. Commercial ELISA kits were used for the determination of IL-6, IL-10 (Peprotech, Rocky Hill, NY) and TNF-α (R&D Systems, Minneapolis, MN) and the detection was performed according to manufacturer’s instructions.

To analyze the impact of MCL and DCIR on T cell stimulation, OT-II T cells were purified from spleen by MACS using the Pan T Cell Isolation Kit II (Miltenyi Biotec, Bergisch Gladbach, Germany) according to the manufacturer’s instructions. 3×10^4^ BMMs or 1.5×10^4^ BMDCs were pulsed with 30 µg/mL ovalbumin (OVA, Hyglos, Bernried am Starnberger See, Germany) in the presence of different dilutions of faecal samples and co-cultivated with 1×10^5^ OT-II T cells in a round-bottom 96 well plate. 10 µg/mL of OVA_323–339_ was used as positive control. After 72 h, T cell activation was analyzed by determination of IL-2 and IFN-γ in the cell culture supernatant using ELISA development kits (Peprotech).

### Ethics statement

Animal experiments were performed in strict accordance with the German regulations of the Society for Laboratory Animal Science and the European Health Law of the Federation of Laboratory Animal Science Associations. The protocol, including the definition of strict humane endpoints, was approved by the Landesamt für Gesundheit und Soziales Berlin (Permit No. G0052/10). All efforts were made to minimize suffering.

### Animals

C57BL/6-Tg (TcraTcrb) 425Cbn/J mice ( = OT-II mice), CLR gene-deficient mouse lines and the respective C57BL/6 J control mice were housed and bred in the animal facility of the Federal Institute for Risk Assessment (BfR) in a temperature- and humidity-controlled room under specific pathogen-free conditions. Food and water were provided *ad libitum*. DCIR^−/−^ (031932-UCD, DCIR KO/Mmcd) mice and MCL^−/−^ (031935-UCD, MCL KO/Mmcd) mice were obtained from the National Institutes of Health-sponsored Mutant Mouse Regional Resource Center (MMRRC) National System. Genotyping of the MCL or DCIR gene in wild-type and MCL- or DCIR-deficient mice was performed using a protocol provided by the Consortium for Functional Glycomics (Figure S1).

### DSS-induced colitis

All DCIR^−/−^, MCL^−/−^ and C57BL/6 J mice used in the experiment were female, between 8 and 10 weeks old and had a body weight of around 20 g at the beginning of the study. Five or six mice of different strains were randomly put together in the same cage at least two weeks before the experiment to ensure similar conditions for mice of different groups. For colitis induction, 3% DSS (w/v) (reagent grade, 35–50 g/mol; MP Biomedicals, Illkirch, France) was added to the drinking water for seven consecutive days. The body weight was recorded daily. On day seven, mice were sacrificed and a disease activity index (DAI) was assessed for each mouse in a blinded manner. The scoring system with scores of 0–4 was based on stool consistency, blood presence in stool and weight loss as described in previous colitis studies [21], [22], modified from [42] (Table 1). Blood in faeces was detected with Greegor’s modified Guaiak test (Haemoccult, Beckman Coulter, Galway, Ireland) according to the manufacturer’s instructions. The colon of each mouse was prepared and its length was measured since colon contraction is a characteristic feature of colitis [43]. Subsequently, the colon was either fixed in 4% buffered formalin for histological analysis or flushed with PBS, snap-frozen in liquid nitrogen and stored at –80°C for later cytokine determination or mRNA expression analysis.

**Table 1:** Scoring for assessment of the disease activity index.

Score	Blood in faeces	Stool consistency	Weight loss (%)
0	No blood detected	Normal	0
1			1–3
2	Blood detected	Loose stool	3–6
3			6–9
4	Gross blood detected	Diarrhea	>9

### mRNA expression analysis by semi-quantitative RT-PCR

For the detection of MCL and DCIR mRNA levels in the colon of untreated and DSS-treated C57BL/6 J mice, total RNA was isolated using the Tri Reagent solution (Life technologies). 2 mL of the solution was added to each frozen colon and samples were incubated at RT for 5 min. Colon tissues were subsequently homogenized using an IKA T10 homogenizer (IKA-Werke GmbH, Staufen, Germany). Cells were further lysed by passing the sample through a 20-gauge needle using a 1 mL syringe. After removal of cell debris by centrifugation at 12000 g and 4°C for 10 min, RNA isolation was performed according to the manufacturer’s instructions. 1 µg of purified RNA was then applied to DNase I digestion (NEB, Frankfurt am Main, Germany) followed by cDNA synthesis using AMV reverse transcriptase (NEB). At this step, a negative control was included for each sample where no reverse transcriptase was added. 1 µL of cDNA per sample was used for DNA amplification by PCR using specific primers for MCL (fw 5′-TCATTACTTTTTACGCTGGA-3′, rev 5′-ACAAATCCTTCTCACCTCAAAG-3′), DCIR (fw 5′-GCTACTTCTCCTGCTGCTGG-3′, rev 5′-TCCAGTCTTCCAACGGTAAA-3′), β-actin (fw 5′-GTCGTACCACAGGCATTGTGATGG-3′, rev 5′-GCAATGCCTGGGTACATGGTGG-3′). As a positive control, cDNA from BMMs (for MCL) or from BMDCs (for DCIR) was used. For visualization of DNA bands, DNA-Dye NonTox (AppliChem, Darmstadt, Germany) was added to the PCR products and samples were loaded on a 1.5% agarose gel.

### Histological analysis of colon sections

Histological analyses were performed as previously described [22]. Briefly, each mouse colon was fixed with 4% buffered formalin and embedded in paraffin. Sections of 4 µm thickness were prepared and stained with hematoxylin and eosin (H&E). Subsequently, they were examined in a blinded manner by an experienced histologist. Each colon was divided into three segments of identical length and each part was graded according to the degree of leukocyte infiltration and mucosal damage (Table 2).

**Table 2:** Scoring for the histological evaluation of intestinal lesions.

Score	Infiltration of inflammatory cells	Mucosal erosion/ulceration
0	None	None
1	Mild	Mild
2	Moderate	Moderate
3	Severe	Severe

### Determination of colonic cytokines

For cytokine determination, thawed colons were homogenized using an IKA T10 homogenizer after addition of a modified Greenberger lysis buffer containing 300 mM NaCl, 15 mM Tris, 2 mM MgCl_2_, 0.5% Triton X-100, protease-inhibitor X, and protease-inhibitor HP (Serva Electrophoresis, Heidelberg, Germany). Protein concentration was determined with the Pierce BCA Protein Assay Kit (Thermo Scientific, Rockford, IL) with BSA as a standard. The concentrations of the cytokines IL-6, IL-1β, TNF-α, IL-10, and IL-12p70 in colon homogenates were determined by cytometric bead array (BD Biosciences) following manufacturer’s instructions. Samples were measured using a FACS Canto II flow cytometer (BD Biosciences) and data were analyzed with the FCAP Array software (BD Bioscience).

### Statistical analysis

Statistical analyses were performed with Mann-Whitney’s U test for all *in vivo* data. *In vitro* data were analyzed with unpaired Student’s t test. Data analysis was performed using the Prism software (GraphPad Software, La Jolla, CA). A *p*-value of *p*<0.05 was considered statistically significant.

## Results

### Binding of MCL and DCIR to commensal microbiota

**Figure 1:**
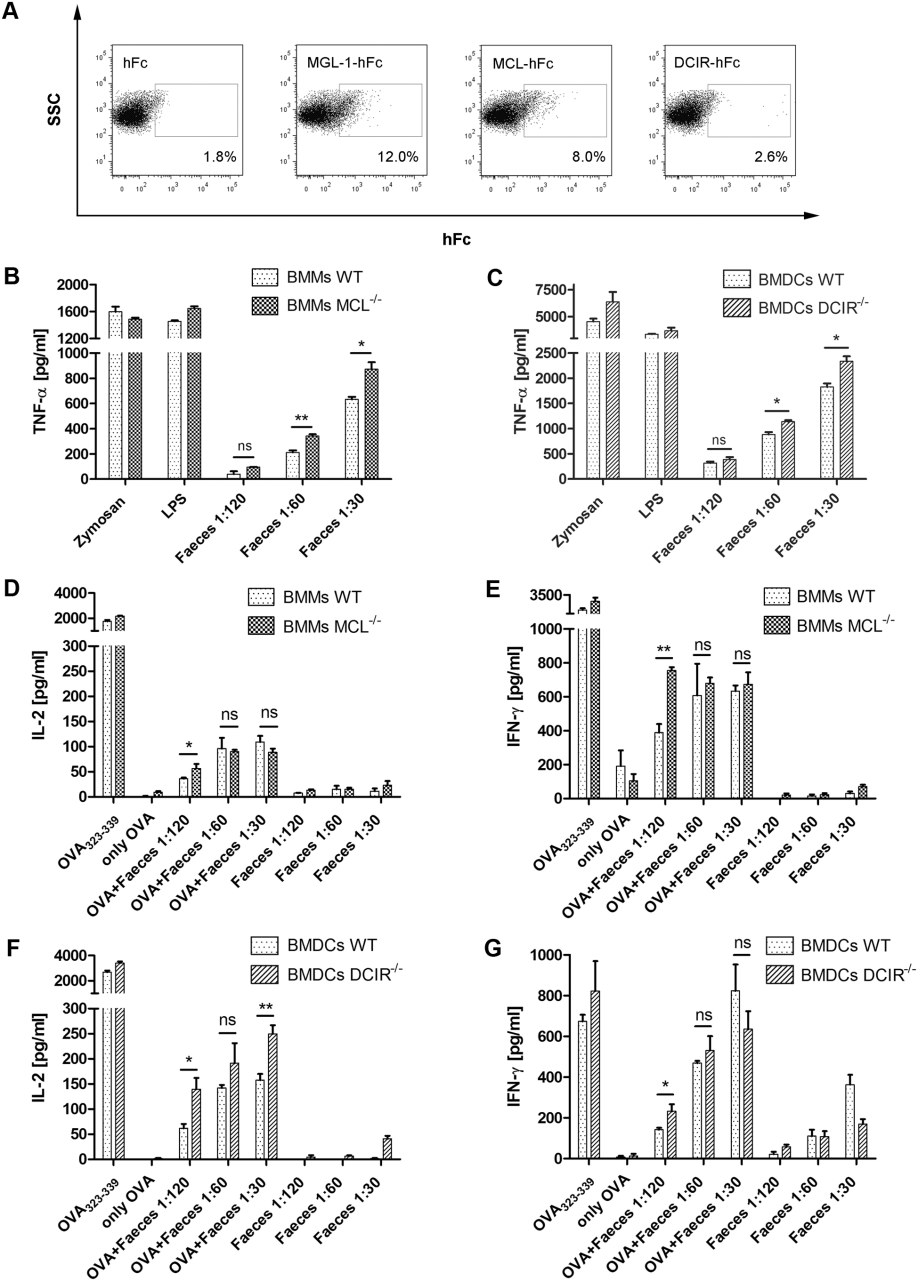
MCL and DCIR recognize commensal intestinal microbiota and modulate APC and T cell cytokine production. Binding of MCL- and DCIR-hFc fusion proteins to stained gut microbes was analyzed by flow cytometry. (**A**) Representative dot plots of one binding experiment with MCL- and DCIR-hFc, with hFc as negative control, and with MGL1-hFc as positive control. Gating and frequencies indicate binding events of CLR-hFc fusion proteins to commensal microbiota. For analysis, it was first gated on Syto 61 positive events ( = stained microbiota) followed by gating on PE positive events ( = CLR-Fc fusion proteins). Data are representative of three independent experiments (triplicates each). (**B**) MCL^−/−^ and wild-type BMMs or (**C**) DCIR^−/−^ and wild-type BMDCs were stimulated with various concentrations of heat-killed gut microbiota, LPS or coated zymosan for 18 h (triplicates each). TNF-α levels in the culture supernatants were determined by ELISA. TNF-α production was significantly increased for MCL^−/−^ BMMs and DCIR^−/−^ BMDCs compared to wild-type APCs. Data are representative of three independent experiments. For analysis of T cell activation, purified OT-II transgenic T cells were co-cultivated with BMMs or BMDCs in the presence of heat-killed gut microbiota and 30 µg/mL OVA for 72 h. (**D**) IL-2 and (**E**) IFN-γ levels were determined in the culture supernatants of stimulated MCL^−/−^ and wild-type BMMs. Similarly, (**F**) IL-2 and (**G**) IFN-γ levels were analyzed in the culture supernatants of stimulated DCIR^−/−^ and wild-type BMDCs. Data are representative of three independent experiments (triplicates each) and are expressed as mean + SEM. The *p*-values were determined with unpaired Student’s t-test (*p<0.05, **p<0.01). Significance is indicated by asterisks (*), ns = no significance.

The interaction of certain CLRs with commensal intestinal microbes has been shown in previous studies [19], [22]. To analyze whether MCL and DCIR interact with murine intestinal commensals, MCL- and DCIR-hFc fusion proteins were generated as previously reported [38] and used for binding studies. The CLR-hFc fusion proteins were incubated with heat-killed intestinal microbes and binding was analyzed by flow cytometry using a fluorescently labeled anti-hFc antibody. MGL1-hFc was used as positive control since binding of this CLR to commensal microbiota was shown in a previous study [20]. As expected, hFc alone did not bind to intestinal microbiota whereas MGL1-hFc exhibited substantial binding to commensal microbiota (Figure 1A). MCL-Fc also recognized a considerable portion of intestinal microbes (Figure 1A). DCIR-Fc only bound to a marginal portion of commensal microorganisms, still DCIR-Fc binding was significantly increased compared to the binding of hFc alone (*p = 0.031, Figure 1A, and data not shown). Co-staining experiments with the lectin WGA that binds to GlcNAc moieties indicated that a minor portion of WGA^+^ microbiota was recognized by MCL-Fc (data not shown). For instance, GlcNAc moieties are present in the cell wall of gram-positive bacteria species [41]. The nature of the MCL ligand remains to be determined in future studies.

### Microbiota recognition by MCL and DCIR modulates TNF-α production by APCs

The binding studies with the MCL- and DCIR-hFc fusion proteins and murine faeces indicate a direct interaction between MCL and, to a low extent, DCIR with intestinal commensal microbes. To analyze the impact of microbiota stimulation on APC effector functions, BMMs or BMDCs from wild-type and MCL^−/−^ or DCIR^−/−^ mice were incubated with heat-killed microbiota of a murine faeces suspension. MCL is predominantly expressed by macrophages [25], [26] whereas DCIR is mainly expressed by immature DCs [32], [36]. Consequently, BMMs from MCL^−/−^ or C57BL/6 control mice and BMDCs from DCIR^−/−^ or C57BL/6 control mice were stimulated with heat-killed microbiota. The stimulation experiments revealed a significantly higher TNF-α production by MCL^−/−^ BMMs upon microbiota stimulation compared to wild-type BMMs (Figure 1B) whereas stimulation with the positive controls zymosan or LPS led to similar TNF-α levels. When BMDCs from DCIR^−/−^ or wild-type mice were stimulated with heat-killed faeces suspensions, a significantly higher TNF-α production by DCIR^−/−^ BMDCs was observed (Figure 1C), particularly at higher faeces concentrations. In tendency, the same effect as for TNF-α was observed for IL-6 levels (data not shown). However, IL-10 production did not differ between wild-type and CLR-deficient APCs (Figure S2). These findings indicate that the recognition of microbiota by MCL and DCIR correlates with a modulated pro-inflammatory cytokine production by APCs.

### Impact of microbiota recognition by MCL and DCIR on T cell activation

To analyze whether the differential microbiota recognition by wild-type and CLR-deficient APCs impacted subsequent T cell activation, APCs from MCL^−/−^, DCIR^−/−^ and wild-type mice were pulsed with the model antigen OVA in the presence of various concentrations of heat-killed intestinal microbiota. Then, OT-II T cells whose TCR is specific for the OVA_323–339_ peptide presented by the MHC-class-II molecule I-A^b^ were added [44]. Cytokine analysis revealed an increased production of IL-2 and IFN-γ when OT-II T cells were co-cultivated with MCL^−/−^ BMMs in the presence of low faeces concentrations (Figure 1D, E). Whereas the IL-2 production by OT-II T cells co-cultured with DCIR^−/−^ BMDCs in the presence of OVA/microbiota was significantly increased (Figure 1F), no marked difference was observed for IFN-γ levels (Figure 1G). These findings indicate that MCL and DCIR not only affect APC functions, but may also modulate subsequent T cell responses.

### Limited role for MCL and DCIR in DSS colitis

**Figure 2:**
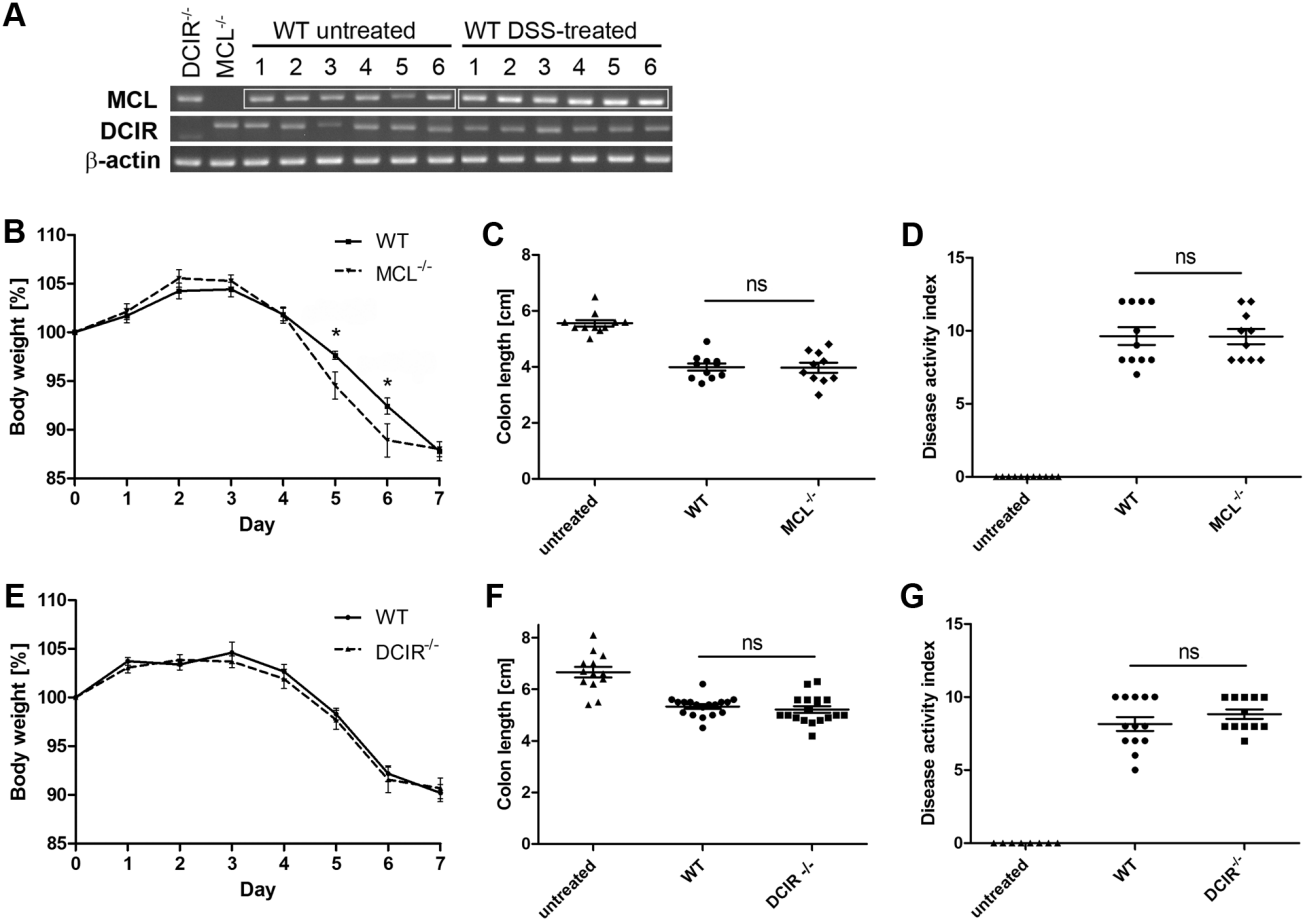
Clinical symptoms of DSS-induced colitis in MCL^−/−^ and DCIR^−/−^ mice. Colitis was induced in wild-type, MCL^−/−^ and DCIR^−/−^ mice by adding 3% DSS into the drinking water for seven consecutive days. (**A**) MCL and DCIR mRNA levels were analyzed by semi-quantitative RT-PCR in colon of untreated and DSS-treated wild-type mice (6 mice per group). As a control, RNA was also isolated from colon of untreated MCL^−/−^ or DCIR^−/−^ mice. β-actin expression levels were determined in all samples to ensure the same quantity and quality of cDNA. MCL mRNA expression in colon of DSS-treated mice was increased compared to the colon of untreated mice while DCIR mRNA levels did not differ between untreated and DSS-treated mice. Disease severity was determined by the daily body weight loss for MCL^−/−^ mice (n = 11) (**B**), and for DCIR^−/−^ mice (n = 18) (**E**), the colon length for MCL^−/−^ mice (**C**), and for DCIR^−/−^ mice (**F**), and a disease activity index (DAI) based on blood occurrence, stool consistency and body weight loss for MCL^−/−^ mice (n = 11 for wild-type and n = 10 for MCL^−/−^ mice) (**D**), and for DCIR^−/−^ mice (n = 13 for wild-type and n = 12 for DCIR^−/−^ mice) (**G**). Data shown are the combined results of two (MCL) or three (DCIR) independent experiments. On day six, one MCL^−/−^ mouse had to be euthanized due to the defined humane endpoints. MCL^−/−^ mice displayed an increased body weight loss compared to wild-type mice, with a significant difference on day 5 and 6. The colon length and DAI did not vary between MCL^−/−^ and wild-type mice. No significant difference in body weight, colon length, and DAI between DCIR^−/−^ and wild-type mice was observed. Data are expressed as mean + SEM. The *p*-values were determined with Mann-Whitney’s U test (*p<0.05). Significance is indicated by asterisks (*), ns = no significance.

To determine the role of the CLRs MCL and DCIR in intestinal immunity, the murine DSS colitis model was employed. It is commonly used to mechanistically investigate the contribution of innate immunity to colitis pathogenesis since adaptive immunity plays a minor role in this model [12]. DSS is toxic to gut epithelial cells and leads to disruption of the mucosal barrier [11], thus it promotes a direct interaction between commensal microbes and APCs in the gut. MCL and DCIR mRNA expression analysis by semi-quantitative RT-PCR revealed increased MCL mRNA levels in the colon of DSS-treated wild-type mice compared to untreated mice (Figure 2A). In contrast, the local DCIR mRNA levels were not altered during DSS colitis (Figure 2A). During experimental colitis, MCL^−/−^ mice exhibited a significantly increased weight loss compared to wild-type mice on day 5 and 6 (Figure 2B) indicating a protective role for MCL. Clinical symptoms or weight loss were not present in untreated MCL^−/−^, DCIR^−/−^ or wild-type mice (data not shown). On day six, one MCL^−/−^ mouse had to be taken out of the experiment due to a too severe weight loss compared to untreated control mice. Since acute colitis leads to colon shortening, the length of each colon was determined on day seven. Indeed, colon length was significantly reduced in all mice treated with DSS compared to untreated mice (Figure 2C). Additionally, a disease activity index (DAI) was assessed in a blinded manner based on stool consistency, the presence of occult blood and weight loss (Table 1). However, no difference in the DAI as well as in the colon length between MCL^−/−^ and wild-type mice was detected (Figure 2C, D) suggesting a limited role for MCL in murine colitis. No difference in body weight loss between DCIR^−/−^ mice and wild-type mice was observed (Figure 2E). Furthermore, no significant difference in colon length (Figure 2F) or DAI (Figure 2G) occurred between DCIR^−/−^ and wild-type mice during DSS colitis. These findings suggest that DCIR might be dispensable for intestinal immunity in this model of experimental colitis.

### Histological analysis of colon sections

**Figure 3:**
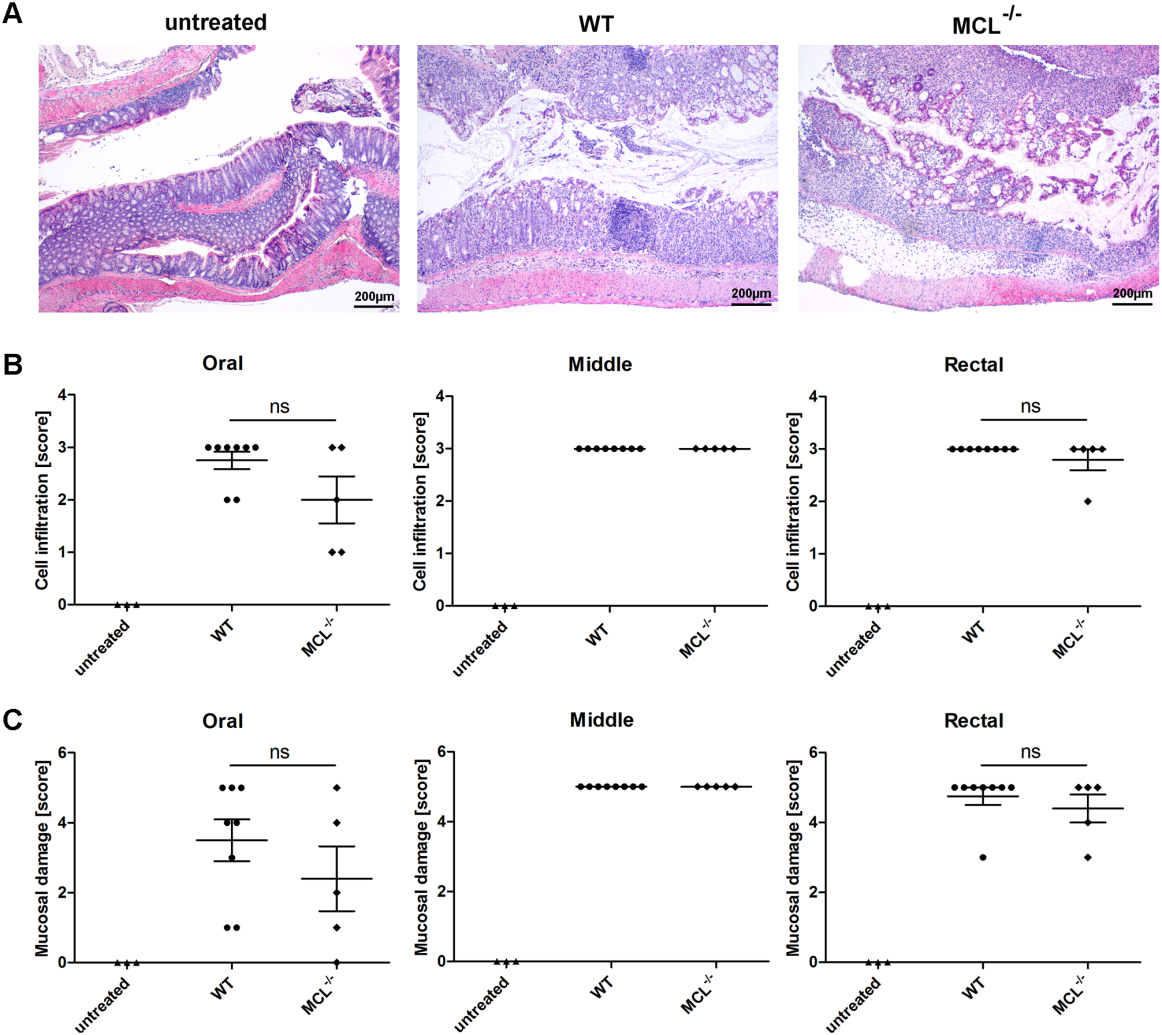
Histological analysis of colon sections from wild-type and MCL^−/−^ mice. Paraffin sections of the colon from untreated or 3% DSS-treated wild-type and MCL^−/−^ mice were prepared at day seven and were stained with hematoxylin and eosin (H&E) for histological evaluation in a blinded manner. Each colon was divided into three segments of identical length (oral, middle, rectal) which were separately analyzed. (**A**) Representative images of paraffin-embedded sections of the rectal part of the colon are shown (40x magnification). The degree of leukocyte infiltration (**B**) and mucosal erosion/ulceration (**C**) was graded from none (score 0) to mild (score 1), moderate (score 3), or severe (score 4). Data are expressed as mean + SEM (n = 8 for wild-type and n = 5 for MCL^−/−^ mice). The *p*-values were determined using Mann-Whitney’s U test. Significance is indicated by asterisks (*), ns = no significance.

**Figure 4:**
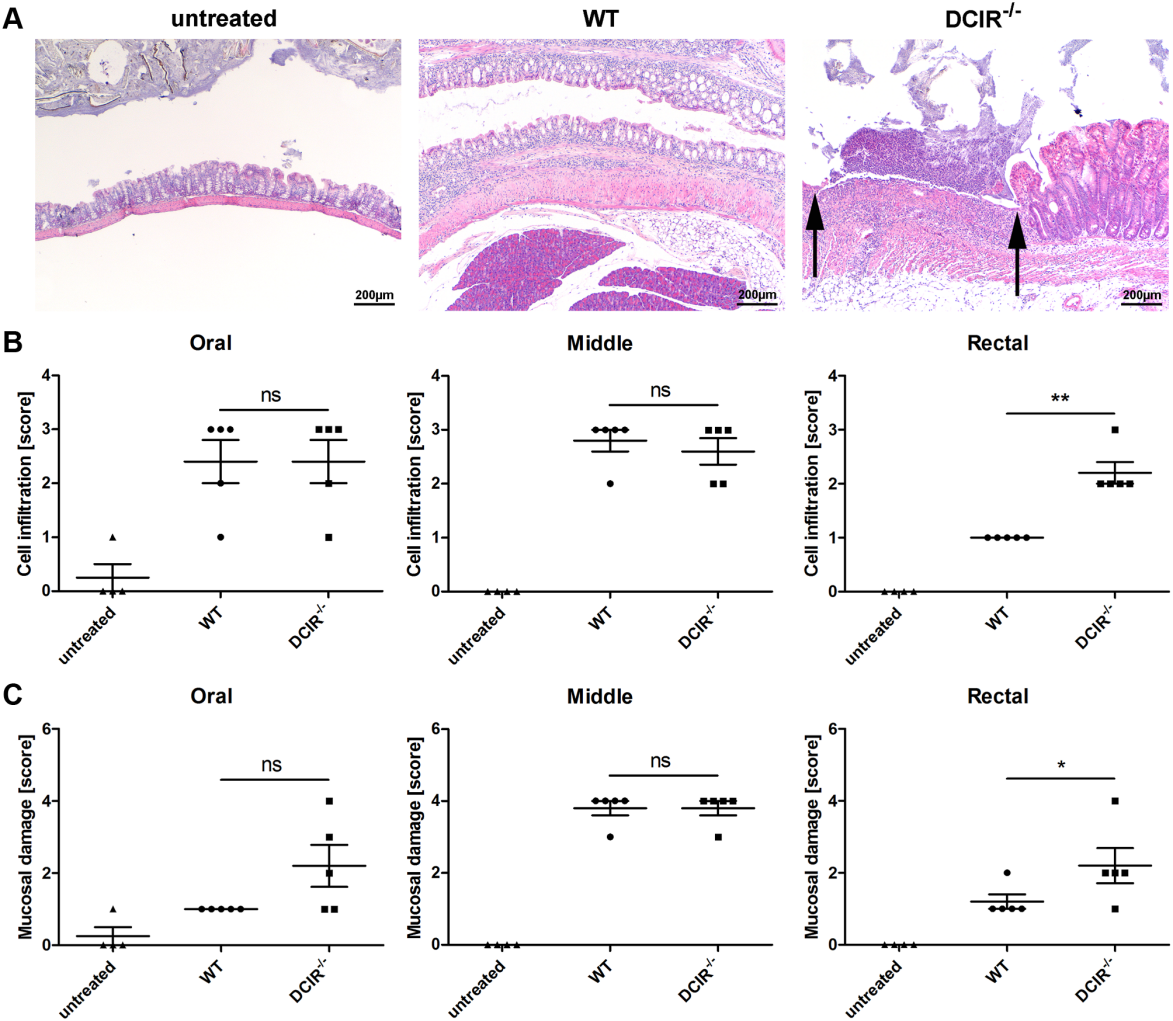
Histological analysis of colon sections from wild-type and DCIR^−/−^ mice. Paraffin sections of the colon from untreated or 3% DSS-treated wild-type and DCIR^−/−^ mice were prepared at day seven and were stained with hematoxylin and eosin (H&E) for histological evaluation in a blinded manner. (**A**) Representative images of paraffin-embedded sections of the rectal part of the colon are shown (40x magnification). Arrows indicate a severe ulcer in the colon from DCIR^−/−^ mice. Each colon was divided into three segments of identical length (oral, middle, rectal) which were separately analyzed. The degree of leukocyte infiltration (**B**) and mucosal erosion/ulceration (**C**) was graded from none (score 0) to mild (score 1), moderate (score 3), or severe (score 4). The scores for both, cell infiltration as well as mucosal ulceration in the rectal part of the colon from DCIR^−/−^ mice were significantly increased compared to wild-type mice. Data are expressed as mean + SEM (n = 5). The *p*-values were determined using Mann-Whitney’s U test (*p<0.05, **p<0.01). Significance is indicated by asterisks (*), ns = no significance.

For further analysis of the grade of disease, a representative number of colon sections from untreated mice and DSS-treated mice were used for histological evaluation. Scores for different grades of leukocyte infiltration and mucosal damage (Table 2) were applied in a blinded manner. During acute colitis, mucosal inflammation causes colonic lesions, ulcers, and bleeding. Thus, granulocytes infiltrating into the lamina propria and submucosa are characteristic features of IBD. Mucosal damage was assessed by analyzing structural changes of the mucosa such as an irregular surface or distortion of the crypt architecture which also includes fibrosis or crypt abscesses. For histological analysis, colon sections were stained with hematoxylin and eosin (Figure 3A). Scores for cell infiltration as well as mucosal ulceration were the same for MCL^−/−^ and wild-type mice (Figure 3B, C). Thus, MCL does not impact leukocyte infiltration into the colon and mucosal damage during experimental colitis. Histological analysis of colon samples from DCIR^−/−^ mice revealed more severe ulcers in the rectal part of the colon of DCIR^−/–^ mice compared to wild-type mice (Figure 4A). This finding was manifested by a significantly higher score for leukocyte infiltration as well as for mucosal damage in the rectal part of the colon of DCIR-deficient mice compared to wild-type mice (Figure 4B, C). In tendency, the same difference was observed for mucosal ulceration in the oral part of the colon (Figure 4C). These findings indicate that DCIR moderately impacts mucosal damage and leukocyte infiltration during acute colitis though the clinical colitis symptoms were similar in DCIR^−/−^ and wild-type mice (ref. Figure 2).

### Local cytokine concentrations in the colon

**Figure 5:**
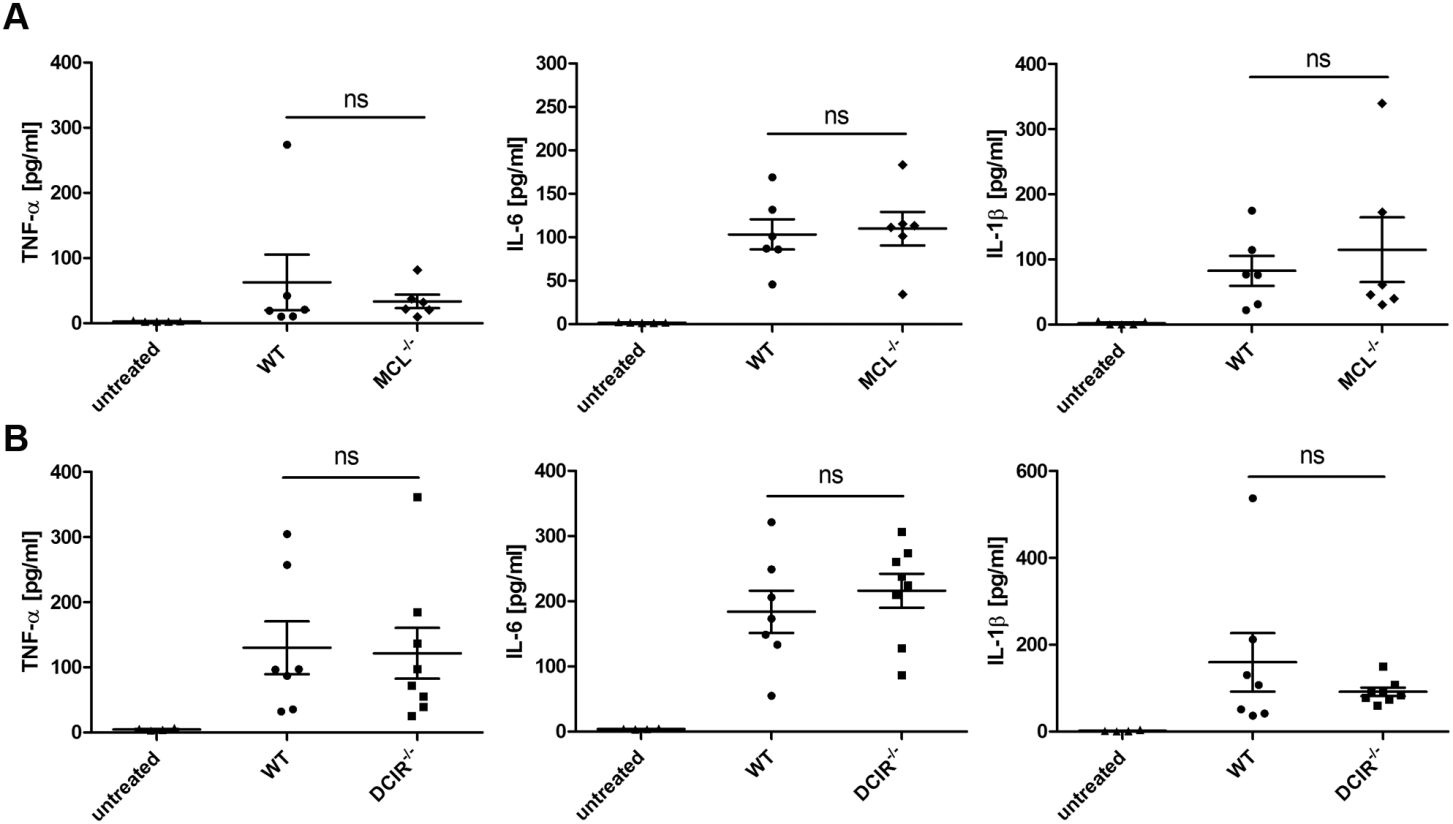
Local cytokine concentrations in the colon of wild-type, MCL^−/−^, and DCIR^−/−^ mice. Colons from untreated wild-type mice or from wild-type and MCL^−/−^ mice (n = 6) (**A**), or wild-type and DCIR^−/−^ mice (n = 7 for wild-type and n = 8 for DCIR^−/−^ mice) (**B**) treated with 3% DSS for seven consecutive days were homogenized and used for cytokine determination by cytometric bead array. Data are expressed as mean + SEM. Significance is indicated by asterisks (*), ns = no significance.

Local cytokine concentrations in the colon were measured during the acute phase of DSS colitis. However, no difference in the levels of the pro-inflammatory cytokines TNF-α, IL-6 and IL-1β was observed between MCL^−/−^ or DCIR^−/−^ and wild-type mice, respectively (Figure 5). Moreover, the concentration of the immune regulatory cytokine IL-10 was similar in DSS-treated wild-type, MCL-, and DCIR-deficient mice (data not shown). Thus, local cytokine levels did not differ between MCL^−/−^ and DCIR^−/−^ mice compared to wild-type mice. In summary, the DSS colitis studies suggest a minor role for MCL and DCIR in regulating colon inflammation during experimental colitis.

## Discussion

In the present study, we analyzed the role of the CLRs MCL and DCIR in murine experimental colitis. As members of the PRR family, CLRs play a crucial role in the recognition of pathogens [45]. They are mainly expressed by APCs and generally bind to carbohydrates present on self or foreign antigens [16], [17]. During IBD, the mucosal barrier in the intestine becomes disrupted followed by an aberrant interaction between commensal intestinal microbes and epithelial cells as well as local immune cells [43]. DCIR has already been shown to recognize self and foreign antigens [46], and to play a crucial role in maintaining the homeostasis of the immune system during the pathogenesis of autoimmune diseases [47]. It has also recently been reported to be involved in the pathogenesis of experimental cerebral malaria [48]. MCL has been demonstrated to bind to the mycobacterial glycolipid TDM [28], [29] and to play a role in the immune response against mycobacteria and *Klebsiella pneumoniae* infection [28], [31]. Based on these functions of MCL and DCIR in immune homeostasis and microbial recognition, we investigated the role of both CLRs in murine colitis. Indeed, binding studies using CLR-hFc fusion proteins revealed a direct interaction of MCL with a considerable portion of commensal intestinal microbiota. Stimulation of APCs with intestinal microbes and APC/T cell co-cultivation assays indicated a regulatory role of MCL and DCIR in TNF-α production by APCs and in subsequent T cell responses.

To analyze the role of MCL and DCIR in intestinal immunity *in vivo*, the phenotype of MCL- and DCIR-deficient mice was characterized in the DSS colitis model which is a common model to investigate the contribution of innate immunity to IBD pathogenesis [11], [12]. The increased MCL mRNA level in the colon of DSS-treated mice suggests a role of MCL in intestinal immunity. The difference in expression might be due to infiltration of MCL-expressing cells into the colon during experimental colitis or due to MCL up-regulation on APC subsets. However, it was reported that MCL is constitutively expressed in myeloid cells and no up-regulation was observed upon *in vitro* stimulation with TDM [28] rendering an infiltration of MCL^+^ cells into the colon more likely. Furthermore, MCL^−/−^ mice exhibited an increased weight loss compared to wild-type mice, but other clinical symptoms were similar for MCL^−/−^ and wild-type mice. It has been proposed that MCL is an activating receptor which is coupled to the spleen tyrosine kinase (Syk) signaling pathway [30]. A recent study demonstrated that MCL delivers activating signals through an ITAM-containing FcRγ chain [28]. Several activating CLRs have already been described to play a regulatory role in IBD. For instance, Dectin-1 and SIGNR3 modulate immune pathology in chemically induced colitis [19], [22]. In addition to CLRs, NLRs play a crucial role in colitis, as mice deficient for NOD2 exhibited more severe clinical symptoms than wild-type mice during the process of colitis [49]. NOD2 binds to the microbial ligand muramyl dipeptide and activates the NF-kB signaling pathway [50]. However, NOD2 was also reported to promote the secretion of the anti-inflammatory cytokine IL-10 as well as proliferation of regulatory T cells during murine colitis [51].

The characterization of the DCIR^−/−^ mice in the DSS colitis model indicated a minor role for DCIR in murine colitis. DCIR mRNA levels were not altered during experimental colitis, however, DCIR was described to be down-regulated during activation of DCs [36] which might have been compensated during experimental colitis by cell infiltration in the colon. Although no difference was observed in body weight loss and colon length of mice, higher histological scores for leukocyte infiltration and mucosal damage in the rectal part of the colon were detected for DCIR-deficient mice. DCIR has previously been shown to bind to mannose- or fucose-based glycan structures [52]. It also binds to self-antigens present on keratinocytes and to foreign antigens such as the envelope glycoprotein E2 of Hepatitis C virus (HCV) [46], [53]. Recently, DCIR was shown to bind to sialylated intravenous immunoglobulin and to play a role in induction of regulatory T cells during allergic airway inflammation in mice [54]. Hence, DCIR might also recognize glycan structures present on gut antigens although we observed only slight binding of a DCIR-hFc fusion protein to heat-killed intestinal microbes. However, this slight binding might also be due to the glycosylation of the CRD in the DCIR-hFc fusion protein since it was recently shown that DCIR exhibits higher ligand affinity in a deglycosylated form [46], [52]. DCIR is the only classical CLR with an intracellular immunoreceptor tyrosine-based inhibitory motif (ITIM) and plays an inhibitory role in response to pathogens [32], [36]. Furthermore, DCIR was reported as a negative regulator of DC expansion [47]. The modulatory effect of DCIR was markedly seen *in vitro* upon stimulation of BMDCs with heat-killed intestinal microbes. *In vivo*, its immune modulatory role was only observed with regard to leukocyte cell infiltration and mucosal damage in the colon. However, it has to be taken into account that other CLRs might also be involved in the regulation of intestinal inflammation. Thus, compensatory effects might explain the limited role of DCIR in colitis observed in the DSS model.

In conclusion, MCL and DCIR modulate TNF-α production upon stimulation of APCs with heat-killed microbiota but play a limited role in intestinal immunity during the pathogenesis of DSS-induced colitis. Further studies are needed to investigate the impact of multiple CLRs in intestinal immunity and potential compensatory mechanisms.

## Supporting Information

Figure S1 **Genotyping of DCIR^−/−^ and MCL^−/−^ mice by PCR.** **(A)** Genotyping of the DCIR gene in wild-type and DCIR^−/−^ mice was performed with the following primers (sequences provided from the Consortium for Functional Glycomics): DR 177 (5′-GCCACATGCTCAGCCTTCAG-3′), DR 483 (5′CACTGTGGGACGTTACTGTC-3′), DR 484 (5′-GGACCATTTTCTTCTGCCTAGA-3′). The wild-type band has a size of 748 bp, while the DCIR knockout band is 457 bp. Shown is the representative analysis of genomic DNA of wild-type (+/+), heterozygous (+/−) and DCIR deficient (−/−) mice. **(B)** Genotyping of the MCL gene in wild-type and MCL^−/−^ mice was performed with the following primers (sequences provided from the Consortium for Functional Glycomics): MC.612 (5′-GTATAATGTATGCTATACGAAGTTATCTCGAG-3′), MC.613 (5′-CTGAAAAAACTTATTGCTCATAATTTACACAGTAT-3′), MC.614 (5′-GGAGGCTTTGGGAGCACATG-3′). The wild-type band has a size of 550 bp, while the MCL knockout band is 360 bp.
(TIF)

Figure S2 **IL-10 production by APCs stimulated with heat-killed commensal intestinal microbiota.** **(A)** MCL^−/−^ and wild-type BMMs or **(B)** DCIR^−/−^ and wild-type BMDCs were stimulated with various concentrations of heat-killed gut microbiota or with LPS as positive control for 18 h (triplicates each). IL-10 levels in the cell culture supernatants were determined by ELISA. Data are representative of three independent experiments and are expressed as mean + SEM. The *p*-values were determined with unpaired Student’s t-test (*p<0.05). Significance is indicated by asterisks (*), ns = no significance.
(TIF)

Checklist S1 **ARRIVE Guidelines Checklist.** The information on the ARRIVE (Animal Research: Reporting *In Vivo* Experiments) guidelines are included in the manuscript as indicated in the checklist.
(PDF)

## References

[1] Kaser A, Zeissig S, Blumberg RS (2010) Inflammatory bowel disease. Annu Rev Immunol 28: 573–621. 20192811 10.1146/annurev-immunol-030409-101225 PMC4620040

[2] Bouma G, Strober W (2003) The immunological and genetic basis of inflammatory bowel disease. Nat Rev Immunol 3: 521–533. 12876555 10.1038/nri1132

[3] Bernstein CN, Shanahan F (2008) Disorders of a modern lifestyle: reconciling the epidemiology of inflammatory bowel diseases. Gut 57: 1185–1191. 18515412 10.1136/gut.2007.122143

[4] Salim SY, Soderholm JD (2011) Importance of disrupted intestinal barrier in inflammatory bowel diseases. Inflamm Bowel Dis 17: 362–381. 20725949 10.1002/ibd.21403

[5] Cerboni S, Gentili M, Manel N (2013) Diversity of pathogen sensors in dendritic cells. Adv Immunol 120: 211–237. 24070386 10.1016/B978-0-12-417028-5.00008-9

[6] Stagg AJ, Hart AL, Knight SC, Kamm MA (2003) The dendritic cell: its role in intestinal inflammation and relationship with gut bacteria. Gut 52: 1522–1529. 12970149 10.1136/gut.52.10.1522 PMC1773829

[7] Abreu MT (2010) Toll-like receptor signalling in the intestinal epithelium: how bacterial recognition shapes intestinal function. Nat Rev Immunol 10: 131–144. 20098461 10.1038/nri2707

[8] Cario E (2010) Toll-like receptors in inflammatory bowel diseases: a decade later. Inflamm Bowel Dis 16: 1583–1597. 20803699 10.1002/ibd.21282 PMC2958454

[9] Strober W, Murray PJ, Kitani A, Watanabe T (2006) Signalling pathways and molecular interactions of NOD1 and NOD2. Nat Rev Immunol 6: 9–20. 16493424 10.1038/nri1747

[10] Rakoff-Nahoum S, Paglino J, Eslami-Varzaneh F, Edberg S, Medzhitov R (2004) Recognition of commensal microflora by toll-like receptors is required for intestinal homeostasis. Cell 118: 229–241. 15260992 10.1016/j.cell.2004.07.002

[11] Wirtz S, Neufert C, Weigmann B, Neurath MF (2007) Chemically induced mouse models of intestinal inflammation. Nat Protoc 2: 541–546. 17406617 10.1038/nprot.2007.41

[12] Wirtz S, Neurath MF (2007) Mouse models of inflammatory bowel disease. Adv Drug Deliv Rev 59: 1073–1083. 17825455 10.1016/j.addr.2007.07.003

[13] Drummond RA, Brown GD (2013) Signalling C-type lectins in antimicrobial immunity. PLoS Pathog 9: e1003417. 23935480 10.1371/journal.ppat.1003417 PMC3723563

[14] Hardison SE, Brown GD (2012) C-type lectin receptors orchestrate antifungal immunity. Nat Immunol 13: 817–822. 22910394 10.1038/ni.2369 PMC3432564

[15] Osorio F, Reis e Sousa C (2011) Myeloid C-type lectin receptors in pathogen recognition and host defense. Immunity 34: 651–664. 21616435 10.1016/j.immuni.2011.05.001

[16] Sancho D, Reis e Sousa C (2012) Signaling by myeloid C-type lectin receptors in immunity and homeostasis. Annu Rev Immunol 30: 491–529. 22224766 10.1146/annurev-immunol-031210-101352 PMC4480235

[17] Lepenies B, Lee J, Sonkaria S (2013) Targeting C-type lectin receptors with multivalent carbohydrate ligands. Adv Drug Deliv Rev 65: 1271–1281. 23727341 10.1016/j.addr.2013.05.007

[18] Yan H, Ohno N, Tsuji NM (2013) The role of C-type lectin receptors in immune homeostasis. Int Immunopharmacol 16: 353–357. 23623943 10.1016/j.intimp.2013.04.013

[19] Iliev ID, Funari VA, Taylor KD, Nguyen Q, Reyes CN, et al (2012) Interactions between commensal fungi and the C-type lectin receptor Dectin-1 influence colitis. Science 336: 1314–1317. 22674328 10.1126/science.1221789 PMC3432565

[20] Saba K, Denda-Nagai K, Irimura T (2009) A C-type lectin MGL1/CD301a plays an anti-inflammatory role in murine experimental colitis. Am J Pathol 174: 144–152. 19095961 10.2353/ajpath.2009.080235 PMC2631327

[21] Saunders SP, Barlow JL, Walsh CM, Bellsoi A, Smith P, et al (2010) C-type lectin SIGN-R1 has a role in experimental colitis and responsiveness to lipopolysaccharide. J Immunol 184: 2627–2637. 20130211 10.4049/jimmunol.0901970

[22] Eriksson M, Johannssen T, von Smolinski D, Gruber AD, Seeberger PH, et al (2013) The C-Type Lectin Receptor SIGNR3 Binds to Fungi Present in Commensal Microbiota and Influences Immune Regulation in Experimental Colitis. Front Immunol 4: 196. 23882266 10.3389/fimmu.2013.00196 PMC3712271

[23] Muller S, Schaffer T, Flogerzi B, Seibold-Schmid B, Schnider J, et al (2010) Mannan-binding lectin deficiency results in unusual antibody production and excessive experimental colitis in response to mannose-expressing mild gut pathogens. Gut 59: 1493–1500. 20682699 10.1136/gut.2010.208348

[24] Seibold F, Konrad A, Flogerzi B, Seibold-Schmid B, Arni S, et al (2004) Genetic variants of the mannan-binding lectin are associated with immune reactivity to mannans in Crohn’s disease. Gastroenterology 127: 1076–1084. 15480986 10.1053/j.gastro.2004.07.056

[25] Balch SG, McKnight AJ, Seldin MF, Gordon S (1998) Cloning of a novel C-type lectin expressed by murine macrophages. J Biol Chem 273: 18656–18664. 9660840 10.1074/jbc.273.29.18656

[26] Arce I, Martinez-Munoz L, Roda-Navarro P, Fernandez-Ruiz E (2004) The human C-type lectin CLECSF8 is a novel monocyte/macrophage endocytic receptor. Eur J Immunol 34: 210–220. 14971047 10.1002/eji.200324230

[27] Lobato-Pascual A, Saether PC, Fossum S, Dissen E, Daws MR (2013) Mincle, the receptor for mycobacterial cord factor, forms a functional receptor complex with MCL and FcepsilonRI-gamma. Eur J Immunol 43: 3167–3174. 23921530 10.1002/eji.201343752

[28] Miyake Y, Toyonaga K, Mori D, Kakuta S, Hoshino Y, et al (2013) C-type lectin MCL is an FcRgamma-coupled receptor that mediates the adjuvanticity of mycobacterial cord factor. Immunity 38: 1050–1062. 23602766 10.1016/j.immuni.2013.03.010

[29] Furukawa A, Kamishikiryo J, Mori D, Toyonaga K, Okabe Y, et al (2013) Structural analysis for glycolipid recognition by the C-type lectins Mincle and MCL. Proc Natl Acad Sci U S A 110: 17438–17443. 24101491 10.1073/pnas.1312649110 PMC3808641

[30] Graham LM, Gupta V, Schafer G, Reid DM, Kimberg M, et al (2012) The C-type lectin receptor CLECSF8 (CLEC4D) is expressed by myeloid cells and triggers cellular activation through Syk kinase. J Biol Chem 287: 25964–25974. 22689578 10.1074/jbc.M112.384164 PMC3406680

[31] Steichen AL, Binstock BJ, Mishra BB, Sharma J (2013) C-type lectin receptor Clec4d plays a protective role in resolution of Gram-negative pneumonia. J Leukoc Biol 94: 393–398. 23709686 10.1189/jlb.1212622 PMC3747124

[32] Kanazawa N (2007) Dendritic cell immunoreceptors: C-type lectin receptors for pattern-recognition and signaling on antigen-presenting cells. J Dermatol Sci 45: 77–86. 17046204 10.1016/j.jdermsci.2006.09.001

[33] Meyer-Wentrup F, Cambi A, Joosten B, Looman MW, de Vries IJ, et al (2009) DCIR is endocytosed into human dendritic cells and inhibits TLR8-mediated cytokine production. J Leukoc Biol 85: 518–525. 19028959 10.1189/jlb.0608352

[34] Meyer-Wentrup F, Benitez-Ribas D, Tacken PJ, Punt CJ, Figdor CG, et al (2008) Targeting DCIR on human plasmacytoid dendritic cells results in antigen presentation and inhibits IFN-alpha production. Blood 111: 4245–4253. 18258799 10.1182/blood-2007-03-081398

[35] Klechevsky E, Flamar AL, Cao Y, Blanck JP, Liu M, et al (2010) Cross-priming CD8+ T cells by targeting antigens to human dendritic cells through DCIR. Blood 116: 1685–1697. 20530286 10.1182/blood-2010-01-264960 PMC2947393

[36] Bates EE, Fournier N, Garcia E, Valladeau J, Durand I, et al (1999) APCs express DCIR, a novel C-type lectin surface receptor containing an immunoreceptor tyrosine-based inhibitory motif. J Immunol 163: 1973–1983. 10438934

[37] Stockinger B, Zal T, Zal A, Gray D (1996) B cells solicit their own help from T cells. The J Exp Med 183: 891–899. 8642293 10.1084/jem.183.3.891 PMC2192359

[38] Maglinao M, Eriksson M, Schlegel MK, Zimmermann S, Johannssen T, et al (2013) A platform to screen for C-type lectin receptor-binding carbohydrates and their potential for cell-specific targeting and immune modulation. J Control Release 175C: 36–42. 10.1016/j.jconrel.2013.12.011 24368301

[39] Schlegel MK, Hütter J, Eriksson M, Lepenies B, Seeberger PH (2011) Defined presentation of carbohydrates on a duplex DNA scaffold. Chembiochem 12: 2791–2800. 22052782 10.1002/cbic.201100511

[40] Eriksson M, Serna S, Maglinao M, Schlegel MK, Seeberger PH, et al (2014) Biological evaluation of multivalent lewis X-MGL-1 interactions. Chembiochem 15: 844–851. 24616167 10.1002/cbic.201300764

[41] Holm C, Jespersen L (2003) A flow-cytometric gram-staining technique for milk-associated bacteria. Appl Environ Microbiol 69: 2857–2863. 12732558 10.1128/AEM.69.5.2857-2863.2003 PMC154518

[42] Cooper HS, Murthy SN, Shah RS, Sedergran DJ (1993) Clinicopathologic study of dextran sulfate sodium experimental murine colitis. Lab Invest 69: 238–249. 8350599

[43] Maloy KJ, Powrie F (2011) Intestinal homeostasis and its breakdown in inflammatory bowel disease. Nature 474: 298–306. 21677746 10.1038/nature10208

[44] Barnden MJ, Allison J, Heath WR, Carbone FR (1998) Defective TCR expression in transgenic mice constructed using cDNA-based alpha- and beta-chain genes under the control of heterologous regulatory elements. Immunol Cell Biol 76: 34–40. 9553774 10.1046/j.1440-1711.1998.00709.x

[45] Hoving JC, Wilson GJ, Brown GD (2014) Signaling C-Type lectin receptors, microbial recognition and immunity. Cell Microbiol 16: 185–194. 24330199 10.1111/cmi.12249 PMC4016756

[46] Bloem K, Vuist IM, van den Berk M, Klaver EJ, van Die I, et al (2013) DCIR interacts with ligands from both endogenous and pathogenic origin. Immunol Lett 158: 33–41. 24239607 10.1016/j.imlet.2013.11.007

[47] Fujikado N, Saijo S, Yonezawa T, Shimamori K, Ishii A, et al (2008) Dcir deficiency causes development of autoimmune diseases in mice due to excess expansion of dendritic cells. Nat Med 14: 176–180. 18204462 10.1038/nm1697

[48] Maglinao M, Klopfleisch R, Seeberger PH, Lepenies B (2013) The C-type lectin receptor DCIR is crucial for the development of experimental cerebral malaria. J Immunol 191: 2551–2559. 23918990 10.4049/jimmunol.1203451

[49] Krishnaswamy JK, Chu T, Eisenbarth SC (2013) Beyond pattern recognition: NOD-like receptors in dendritic cells. Trends Immunol 34: 224–233. 23352728 10.1016/j.it.2012.12.003 PMC3646908

[50] Mo J, Boyle JP, Howard CB, Monie TP, Davis BK, et al (2012) Pathogen sensing by nucleotide-binding oligomerization domain-containing protein 2 (NOD2) is mediated by direct binding to muramyl dipeptide and ATP. J Biol Chem 287: 23057–23067. 22549783 10.1074/jbc.M112.344283 PMC3391102

[51] Macho Fernandez E, Valenti V, Rockel C, Hermann C, Pot B, et al (2011) Anti-inflammatory capacity of selected lactobacilli in experimental colitis is driven by NOD2-mediated recognition of a specific peptidoglycan-derived muropeptide. Gut 60: 1050–1059. 21471573 10.1136/gut.2010.232918

[52] Bloem K, Vuist IM, van der Plas AJ, Knippels LM, Garssen J, et al (2013) Ligand binding and signaling of dendritic cell immunoreceptor (DCIR) is modulated by the glycosylation of the carbohydrate recognition domain. PLoS One 8: e66266. 23776650 10.1371/journal.pone.0066266 PMC3679074

[53] Florentin J, Aouar B, Dental C, Thumann C, Firaguay G, et al (2012) HCV glycoprotein E2 is a novel BDCA-2 ligand and acts as an inhibitor of IFN production by plasmacytoid dendritic cells. Blood 120: 4544–4551. 23053572 10.1182/blood-2012-02-413286

[54] Massoud AH, Yona M, Xue D, Chouiali F, Alturaihi H, et al.. (2014) Dendritic cell immunoreceptor: a novel receptor for intravenous immunoglobulin mediates induction of regulatory T cells. J Allergy Clin Immunol 133: 853–863 e855. 10.1016/j.jaci.2013.09.029 24210883

