# Supplementary figures and images for: Role of the C-Type Lectin Receptors MCL and DCIR in Experimental Colitis
Source: PLoS One. 2014 Jul 28;9(7):e103281. doi: 10.1371/journal.pone.0103281 (PMC4113383; doi:10.1371/journal.pone.0103281)

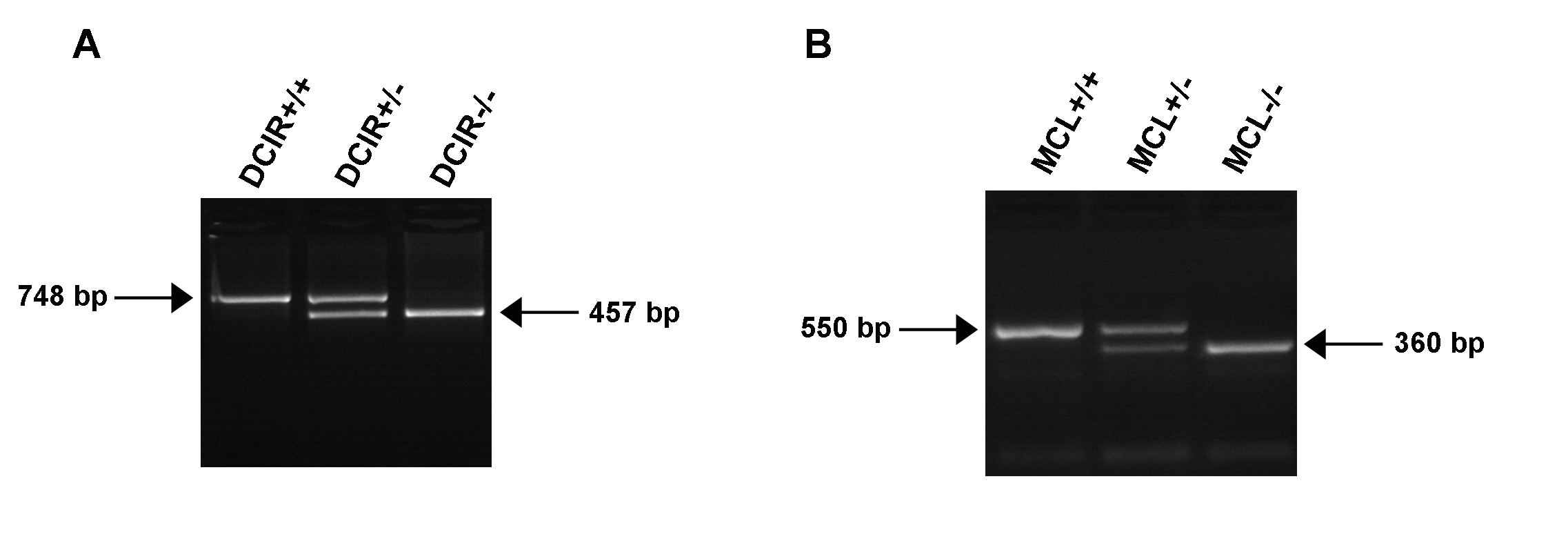

Supplement: Figure S1 — Genotyping of DCIR−/− and MCL−/− mice by PCR. (A) Genotyping of the DCIR gene in wild-type and DCIR−/− mice was performed with the following primers (sequences provided from the Consortium for Functional Glycomics): DR 177 (5′-GCCACATGCTCAGCCTTCAG-3′), DR 483 (5′CACTGTGGGACGTTACTGTC-3′), DR 484 (5′-GGACCATTTTCTTCTGCCTAGA-3′). The wild-type band has a size of 748 bp, while the DCIR knockout band is 457 bp. Shown is the representative analysis of genomic DNA of wild-type (+/+), heterozygous (+/−) and DCIR deficient (−/−) mice. (B) Genotyping of the MCL gene in wild-type and MCL−/− mice was performed with the following primers (sequences provided from the Consortium for Functional Glycomics): MC.612 (5′-GTATAATGTATGCTATACGAAGTTATCTCGAG-3′), MC.613 (5′-CTGAAAAAACTTATTGCTCATAATTTACACAGTAT-3′), MC.614 (5′-GGAGGCTTTGGGAGCACATG-3′). The wild-type band has a size of 550 bp, while the MCL knockout band is 360 bp. (TIF) [file pone.0103281.s001.tif]

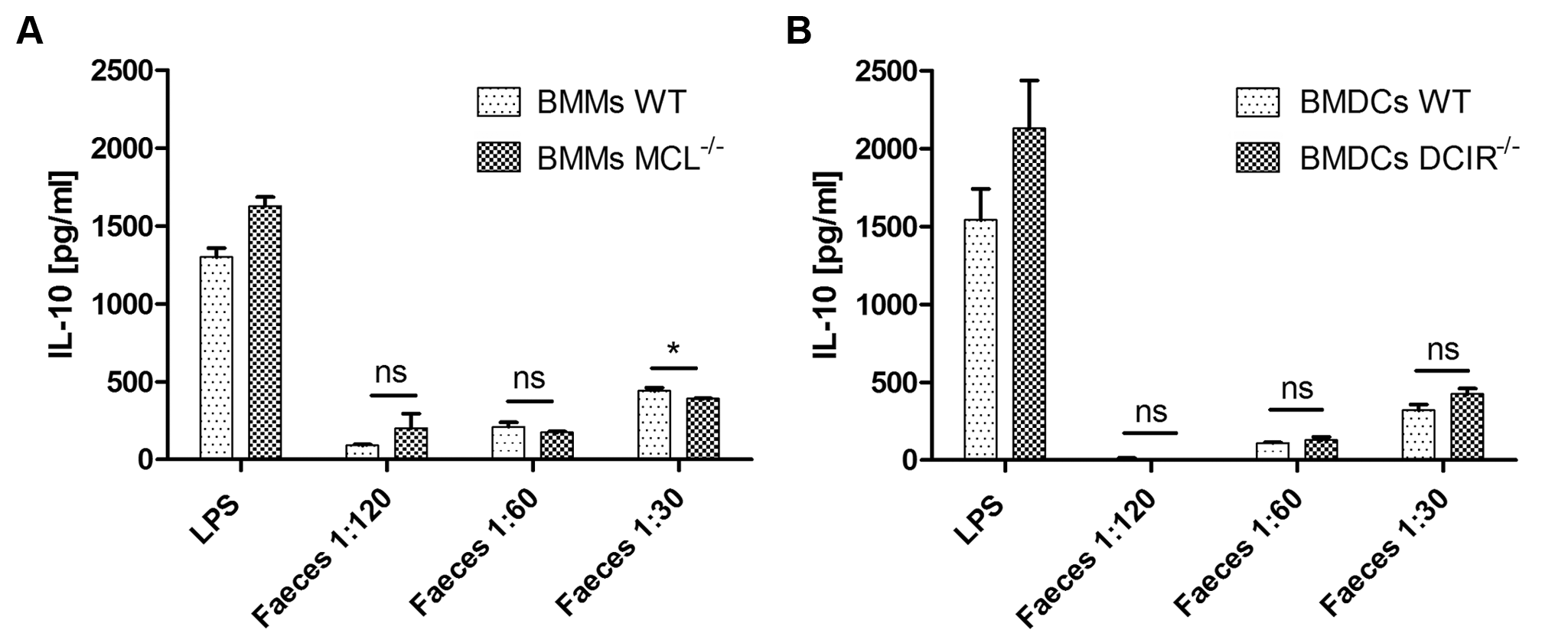

Supplement: Figure S2 — IL-10 production by APCs stimulated with heat-killed commensal intestinal microbiota. (A) MCL−/− and wild-type BMMs or (B) DCIR−/− and wild-type BMDCs were stimulated with various concentrations of heat-killed gut microbiota or with LPS as positive control for 18 h (triplicates each). IL-10 levels in the cell culture supernatants were determined by ELISA. Data are representative of three independent experiments and are expressed as mean + SEM. The p-values were determined with unpaired Student’s t-test (*p<0.05). Significance is indicated by asterisks (*), ns = no significance. (TIF) [file pone.0103281.s002.tif]
